# Measuring Debilitating and Facilitating Anxiety Within Nursing Simulation

**DOI:** 10.3390/nursrep15010001

**Published:** 2024-12-24

**Authors:** Janet M. Reed, Tracy Dodson, Lynette Phillips, Amy Petrinec

**Affiliations:** 1 College of Nursing, Kent State University, Kent, OH 44242, USA; tdodson4@kent.edu (T.D.); apetrine@kent.edu (A.P.); 2 College of Public Health, Kent State University, Kent, OH 44242, USA

**Keywords:** simulation anxiety, debilitating anxiety, facilitating anxiety, nursing, simulation

## Abstract

Background: Anxiety in simulations can be influenced by various factors that either motivate or immobilize students. Understanding simulation anxiety is crucial for educators to design appropriately challenging scenarios without overwhelming students. No instruments have yet been tested to differentiate between debilitating and facilitating anxiety within nursing simulations. Methods: A quantitative repeated measures design was used to examine students’ baseline and pre-simulation anxiety with 90 pre-licensure junior level nursing students. The Achievement Anxiety Test (AAT) was administered to differentiate levels of debilitating and facilitating anxiety. Results: The revised AAT demonstrated preliminary validity and reliability for measuring debilitating and facilitating anxiety when used in nursing simulation. Linear regression showed only debilitating anxiety significantly predicted pre-simulation state anxiety. Baseline anxiety has a significant impact on students, increasing debilitating anxiety in simulated settings. Conclusions: To ensure success in practice settings, it is important to address students baseline anxiety to support a successful transition into practice. This study was prospectively determined to be exempt with the University’s Institutional Review Board on 6 December 2022 and was not prospectively registered in a formal registry.

## 1. Introduction

Generation Z college students experience high levels of anxiety [1] and have even been named “The Anxious Generation” [2]. This particularly holds true for nursing students, who face additional stressors from demanding academics, clinical performance expectations, and simulations [3]. High anxiety can hinder academic success, impact physical and mental health, and diminish effectiveness in healthcare teams [4]. Anxiety related to simulation performance may also impede knowledge transfer to practice, potentially leading to less prepared nurses for complex environments. Anxiety specifically within simulation is complex, nuanced, and can be influenced by a variety of factors that either motivate or paralyze students. While simulation anxiety is often viewed negatively, it can sometimes drive students toward greater motivation and improved performance within the simulation setting [5].

Anxiety can be categorized as state anxiety, a temporary emotional response to specific situations, or trait anxiety, a stable predisposition to perceive situations as threatening [6]. Additionally, anxiety can be classified as debilitating, which impairs performance and focus, or facilitating, which enhances focus and acts as a motivational force [7]. Understanding and measuring the characteristics of simulation anxiety is crucial for educators to develop simulation scenarios with the optimal level of difficulty and challenge, without overwhelming or paralyzing students. While evaluation instruments exist that measure state and trait anxiety, to date, no instrument has been used to differentiate and measure debilitating and facilitating anxiety within the context of nursing simulations. Therefore, this study aimed to conduct preliminary testing of validity and reliability for such a tool within nursing simulations, explore the relationships between levels of debilitating and facilitating anxiety with student demographics and baseline state/trait anxiety, and describe how pre-simulation state/trait anxiety is influenced by these anxiety levels.

Anxiety among college students has increased over time with almost 60% of college students having mild, moderate, or severe anxiety [8]. Over 30% of college students report diagnosis or treatment by a professional for anxiety in the past year [9,10]. For college students seeking a nursing degree, this anxiety is compounded by the rigor of nursing education, with research identifying that nursing students often experience significantly higher anxiety levels than the general college student population [11]. Compounding this problem, highly challenging simulations used throughout nursing education to ensure competent and safe graduate nurses often result in high levels of stress and anxiety which can be distressful and debilitating for some students [12,13]. Although the long-term goal of nursing simulation is to reduce anxiety in future nursing practice [14], simulation often results in high short-term anxiety related to fear of the unknown, lack of experience, technostress, and trepidation about making mistakes in front of others [5]. Teaching students to manage and work through simulation anxiety is an important skill that can carry over into future nursing practice.

Studies attempting to understand the impact of simulation anxiety on learning and performance have shown conflicting results [15]. Interventions for reducing stress and anxiety among nursing students have also shown mixed results as to their effectiveness [16]. Nurse educators may not understand when to use anxiety-reducing measures versus when to push and challenge students out of their comfort zones in simulation. These inconsistent findings suggest that anxiety has traditionally been studied with the assumption that it has a negative influence on outcomes without the alternative perspective that anxiety may be helpful or harmful depending on the individual.

Alpert and Haber’s (1960) theory on the differentiation between facilitating versus debilitating anxiety has been largely studied through the lens of testing anxiety, but it has not yet been tested in nursing simulation. Alpert and Haber (1960) describe facilitating anxiety as motivational because it assists learners in overcoming and completing challenges [7]. By contrast, debilitating anxiety is defined as that which causes learners to avoid an activity to avert the source of anxiety. Cognitive appraisal determines whether anxiety steers the individual toward or away from problem-solving. Cognitive Appraisal Theory [17] provides one theory that can be used to provide theoretical justification for the multifactorial nature of anxiety. The primary appraisal of a stressor involves asking “What’s at stake?” and secondary appraisal asks, “What can be done?” using coping mechanisms as well as emotions and problem-solving [18]. To build a deeper understanding of the phenomena of simulation anxiety within nursing education, it is advantageous to separate anxiety into helpful or harmful types. However, a tool does not yet exist to evaluate anxiety within the context of simulation. One tool that has been used to measure both debilitating and facilitating in the context of test anxiety is the Achievement Anxiety Test (AAT) by Alpert and Haber (1960) [7]. The AAT has been validated over several decades [19] but has not yet been applied to simulation anxiety for nursing students. By separating debilitating anxiety from facilitating anxiety, nurse educators can better guide students to become more resilient nurse graduates. By partitioning out helpful and harmful anxiety, nurse educators can also more easily identify students with high levels of debilitating anxiety and use targeted interventions specifically for these students.

### Study Purpose/Objectives

The overall purpose of this study was to differentiate and describe levels of facilitating and debilitating anxiety within nursing simulations, for a clearer understanding of the impact of anxiety on undergraduate nursing students in simulation. The objectives of this study were to (1) describe the preliminary validity and reliability of a new modified version of Alpert and Haber’s (1960) Achievement Anxiety Test (AAT) for use in nursing simulation to separate facilitating and debilitating anxiety; (2) explore the relationships between the levels of debilitating and facilitating anxiety with student demographics and baseline state/trait anxiety; (3) describe how pre-simulation state/trait anxiety may be affected by the level of facilitating/debilitating anxiety as measured by the AAT.

## 2. Materials and Methods

### 2.1. Design and Sample

This quantitative research study used a repeated measures design with junior-level BSN undergraduate nursing students enrolled in a large medical-surgical nursing course at a public Midwestern University in the fall semester of 2023. Institutional Review Board approval was obtained at the exempt level, and students had no repercussions for not participating in the research. A total of 148 undergraduate junior-level BSN nursing students were invited to participate in the study. A total of 101 students consented to participate in the study (68% response rate) with 11 entries later removed from the analysis due to missing data, leaving a total sample of *n* = 90. All the students participated in the simulation as part of regularly scheduled learning activities; however, only data from those students who consented to participate in the research were included. Student email addresses were collected but then changed to unique numerical identifiers to maintain confidentiality. The theoretical framework used was Alpert and Haber’s [7] theory on facilitating versus debilitating anxiety.

### 2.2. Measures

The Alpert and Haber [7] Achievement Anxiety Test (AAT) is an instrument that measures the levels of facilitating and debilitating anxiety. It was originally created to measure academic test anxiety and has successfully been used in a variety of educational research fields such as foreign language learning [20] and flight simulations [21]. The AAT uses a self-report scale with a 5-point Likert response format with two independent subscales each measuring a separate and opposite construct. One subscale measures facilitating anxiety, and one subscale measures debilitating anxiety, with higher sums representative of higher levels of each type of anxiety. The AAT has 19 questions: nine questions for facilitating anxiety and ten questions for debilitating anxiety [19]. Reliability estimates of AAT range from 0.65–0.88 [22]. Permission to revise the AAT instrument was received to allow for minor wording changes to reflect the nuances of the nursing simulation instead of test anxiety. To achieve content validity of these revisions, an expert panel consisting of six nursing educators with extensive simulation experience in a variety of courses across two separate public universities reviewed the revised AAT for item representativeness. This expert review resulted in an overall content validity index (CVI) of 0.82 for the full scale using the average item CVI for all 19 questions. Additionally, the expert panel gave suggestions for refinement of the wording to improve clarity of items on the AAT for use in nursing simulations.

The State-Trait Anxiety Inventory (STAI) [6] is a widely used tool to measure self-reported state and trait anxiety. However, due to its length (40 items) and to reduce participant fatigue, researchers have developed shorter, reliable versions, such as the five-item short forms by Zsido et al. [19]. State anxiety was measured using the short form STAIS-5, and trait anxiety using STAIT-5 [23]. The psychometric properties of STAIS-5 and STAIT-5 have shown good reliability and internal consistency (Cronbach’s α 0.86–0.91), as well as high correlations with the full STAI (0.88 for trait; 0.86 for state) [23]. Subsequent studies have demonstrated sound psychometric properties in various disciplines including nursing students [24,25].

### 2.3. Procedures

The data collection survey for this study used Qualtrics software (https://www.qualtrics.com/en-au/lp/qualtrics/, access date 9 October 2023). There were two data collection points with students. After consent and demographics were collected, one week before the scheduled course simulation, the students were administered the Alpert and Haber (1960) AAT to measure their baseline levels of facilitating and debilitating anxiety, as well as STAIS-5 and STAIT-5 to measure their baseline levels of state and trait anxiety. The second data collection point occurred one week later when the students arrived at the simulation lab on their scheduled clinical day to participate in a course-required face-to-face high-fidelity manikin nursing simulation. After a thorough pre-briefing and orientation to the simulation environment, guided by the Healthcare Simulation Standards of Best Practice for simulation briefing [26], the students were again administered STAIS-5 and STAIT-5 just before the simulation commenced and prior to simulation role assignment. The students worked in teams of four students with the high-fidelity simulator mannikin for two separate simulations: one on heart failure and one on renal failure, each lasting approximately 30 min. After the simulation ended, the students were debriefed for 30 min using INASCL guidelines and the PEARLS debriefing method [27].

### 2.4. Data Analysis

All the data were assessed for duplication and missing values and analyzed using IBM Statistical Package for the Social Sciences (SPSS) Version 28. Missing data were removed from the analyses using listwise deletion. Descriptive statistics such as means, standard deviations (SD), frequencies, and percentages were analyzed. For the first objective, we used a reliability analysis using coefficient (Cronbach’s) alpha (α) to examine the internal consistency of the scores on the scales for the two data collection time points for both the STAIS-5 and STAIT-5 and the AAT measures with subscales. As the two subscales of the AAT measure opposing constructs and are theoretically inversely correlated [7], Cronbach’s alphas were run separately for each subscale in concordance with reliability testing of previous AAT versions [22]. Exploratory factor analysis (EFA) utilizing principal axis factoring (PAF) with oblique (direct oblimin) rotation was carried out to determine the factor structures of the revised debilitating and facilitating anxiety scales. Principal axis factoring was selected since the goal of the analysis was to determine the underlying factor structure rather than overall item reduction of the scales. A scree plot analysis and eigenvalue ≥1.0 determined the retention of factors. Problematic items of the scales were identified if the primary factor loadings were <0.40 and/or the secondary factor loadings were >0.30. To assess the construct validity of the revised AAT, we employed several strategies including content validity, factorial validity on EFA, and convergent validity in how it correlated with STAIS-5 and STAIT-5.

For study objective two, correlations between the subscales were run to check the alignment with theoretical expectations. Independent samples t-tests were run to examine the changes in anxiety between the student demographic factors such as gender or self-reported history of anxiety disorders. A one-way ANOVA was used to compare means for three or more categories. For study objectives two and three, Pearson’s correlations were calculated to explore the relationships between various types of anxiety. Linear regression analysis was used to evaluate the relationships between the measures and adjust for potential confounders as needed.

## 3. Results

### 3.1. Sample

Within the final sample (*n* = 90), most of the participants were female (91.9%) with 83.3% identifying as white/Caucasian ethnicity (see Table 1). The participants’ ages ranged from 19 to 31, with 90% between the ages of 19 and 22. Most (84%) reported being heterosexual/straight, with 11% bisexual and 3% gay/lesbian. Forty percent of students reported having a past diagnosis of anxiety.

### 3.2. Instrument Reliability

The first objective of this study was to describe the preliminary validity and reliability of a new modified version of Alpert and Haber’s [7] Achievement Anxiety Test (AAT) to separate debilitating and facilitating anxiety within undergraduate nursing simulation. The revised AAT was found to be a reliable measure for a nursing simulation with a Cronbach alpha of 0.71 for the facilitating anxiety subscale, and 0.81 for the debilitating anxiety subscale. The debilitating subscale was significantly moderately negatively correlated with the facilitating subscale (−0.595, *p* < 0.001), aligning with theoretical expectations. The STAIS-5 and STAIT-5 were also found to be reliable instruments with Cronbach alphas of 0.787 (baseline state); 0.773 (baseline trait); 0.828 (pre-simulation state); and 0.826 (baseline trait).

After performing data screening, the EFA of the debilitating anxiety scale yielded a two-factor solution explaining 54% of the variance (KMO = 0.815, Bartlett’s, *p* < 0.001). Items #3 and #17 were found to be problematic due to low primary loading and the only item loading on factor 2, respectively. Table 2 demonstrates the factor loadings of the debilitating anxiety scale. The EFA of the facilitating anxiety scale yielded a 3-factor solution explaining 60% of the variance (KMO = 0.771, Bartlett’s, *p* < 0.001). Items #6, #15, and #18 were problematic due to significant cross loading on other factors or not loading (no significant correlation for #15) on any factors as shown in Table 3.

### 3.3. Relationships Among Variables

The second objective was to explore the relationships between the levels of debilitating and facilitating anxiety with student demographics as well as baseline state and trait anxiety within simulation. The females (*n* = 82) had higher mean debilitating anxiety (33.01) than the males (*n* = 6, 29.33) or non-binary participants (n = 2, 30.50). Additionally, the females had lower mean facilitating anxiety (24.72) than the males (26.50) or non-binary participants (26.00). However, these differences were not statistically different from each other based on Welch’s ANOVA test for unequal variances. Significant differences in the anxiety types were found between the students with an anxiety diagnosis compared to those with no anxiety diagnosis for baseline state and trait anxiety measures (Table 4).

### 3.4. Relationships Between Types of Anxiety

The third objective of this study was to describe how pre-simulation state and trait anxiety may be affected by the level of facilitating or debilitating anxiety as measured by the AAT. The baseline state and trait anxiety measures showed low to moderate positive correlations with debilitating anxiety and low to moderate negative correlations with facilitating anxiety (see Table 3). Pre-simulation state and trait anxiety measures were moderately positively correlated with debilitating anxiety and moderately negatively correlated with facilitating anxiety demonstrating construct validity as this is aligned with theoretical expectations (see Table 5).

Multivariable linear regression modeling was performed for each of the two state and trait anxiety measures (baseline and presimulation) as dependent variables. Each model was tested by including the anxiety diagnosis and debilitating and facilitating anxiety variables with a univariable significance below 0.05. Analyses showed that an anxiety diagnosis was associated with a 2.3-point increase in the baseline state anxiety (*p* < 0.001) when controlling for facilitating anxiety and a 1.6-point increase in baseline trait anxiety when controlling for debilitating anxiety (*p* = 0.02). The pre-simulation state and trait anxiety were each associated with debilitating anxiety (b = 0.34, *p* < 0.001 and b = 0.33, *p* < 0.001, respectively) but neither was associated with self-reported anxiety diagnosis or facilitating anxiety.

### 3.5. Summary of Findings

The revised AAT showed preliminary evidence of being a valid and reliable tool for measuring debilitating and facilitating anxiety for nursing simulation, yet future work with a larger sample size is needed to confirm the findings regarding the psychometric properties of the AAT. Gender did not result in significant differences in the type of anxiety. Forty percent of the sample had a past diagnosis of an anxiety disorder, and this corresponded with significant differences in their baseline state and trait anxiety as well as higher overall levels of debilitating anxiety. Only debilitating anxiety was found to significantly predict the amount of pre-simulation state anxiety that the students experienced. This implies that if nurse educators can identify students with debilitating anxiety, targeted interventions may be employed to reduce simulation paralysis which may not only help students be successful in simulation, but also with future clinical practice in stressful situations.

## 4. Discussion

**Figure 1:**
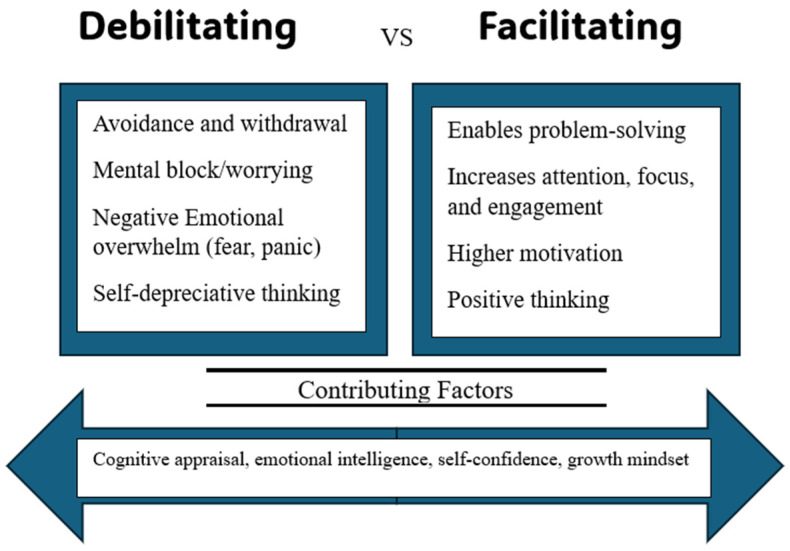
Conceptual factors affecting debilitating and facilitating simulation anxiety.

This study aimed to evaluate the validity and reliability of the revised Achievement Anxiety Test (AAT) for measuring debilitating and facilitating anxiety in nursing simulation contexts, while also exploring how these forms of anxiety relate to student demographics, baseline state and trait anxiety, and the influence of presimulation state and trait anxiety levels. Investigating and understanding the differences between students’ facilitating and debilitating anxiety is a novel approach in nursing education that challenges many longstanding assumptions about anxiety. Relying solely on a debilitating perspective about simulation anxiety oversimplifies the complex nature of anxiety. The findings of the first objective of this study demonstrated that the revised AAT showed preliminary evidence of validity and reliability for differentiating types of anxiety among nursing students before simulation. The exploratory factor analysis suggested that facilitating anxiety is not a single, uniform construct, but rather that it can be broken down into different factors. These factors reflect the different ways anxiety enhances performance, such as motivating, increasing attention, enhancing effort, or boosting focus under pressure (see Figure 1). The 60% variance for the facilitating subscale indicates that more than half of the differences in responses to the facilitating anxiety questions can be understood through these three factors. The ten questions for the debilitating subscale of the revised AAT loaded onto two factors explaining 54% variance. This could represent, for instance, different aspects of debilitating anxiety that impair performance, such as emotional overwhelm (e.g., fear, panic) or cognitive interference (e.g., worrying, distractibility) (see Figure 1).

Our findings for multiple factors within each subscale of the AAT were similar to the findings of Plake [28] who noted five factors within the full AAT with psychology students, demonstrating the multifaceted nature of anxiety. Cognitive Appraisal Theory [17] provides theoretical justification for the multiple factors involved such as cognitive responses, emotionality, and physiologic or somatic anxiety which contribute to the highly individualized nature of anxiety. The emerging factors indicate that anxiety is more complex than originally proposed by Alpert and Haber. These results suggest the need for future revision of the revised AAT for simulation to remove items which did not have primary loading (such as #15) or low loadings or cross loading items. Additionally, some of the problematic items identified by the EFA had overcomplicated wording which might have confused respondents, and so subsequent future revisions will need to incorporate clearer wording. The results of this initial analysis will lead to future revisions of the AAT for measuring debilitating and facilitating anxiety for nursing students in simulation to improve its validity.

By distinguishing between these two types of anxiety, educators can gain deeper insights into the underlying factors influencing student performance and mental well-being during simulation activities. In objective 2 in this study, we examined the relationships and found that facilitating anxiety was moderately negatively correlated with debilitating anxiety, aligning with Alpert and Haber’s [7] theory and supporting construct validity. Being able to use a tool such as the AAT to separate debilitating and facilitating anxiety allows educators to validate the motivational effects of good anxiety and to identify individual sources of debilitating anxiety, such as the fear of failure or lack of confidence.

Recognizing the distinct anxiety types can lead future research to develop targeted interventions to address the causes of debilitating anxiety to better support student learning. For example, debilitating anxiety may be addressed by helping students develop coping strategies such as positive reframing and mindfulness, essential resilience skills for navigating stressful clinical scenarios in future practice [29,30]. By increasing one’s “trait mindfulness”, the cognitive load decreases during simulations leaving more working memory for the tasks at hand [31]. Trait mindfulness can be defined as a heightened awareness and ability to pay attention to the present moment with curiosity, openness, and acceptance and without judgment [31]. The AAT provides a self-evaluative tool for students to reflect on their tendencies toward debilitative or facilitative anxiety and may increase their mindfulness of these concepts.

Facilitating anxiety, though experienced similarly, is fundamentally different in that it drives and motivates students towards better performance. Murphy et al. [32] suggest that with music performance anxiety, it is the underlying emotions and thoughts, rather than its intensity or quantity, that determine whether anxiety is debilitative or facilitative. Nurses often must “perform” with others watching in practice and in simulation, similar to a musician performing. Thoughts and emotions can be trained to be positively reframed in simulation to prevent negative rumination which can lead to debilitating anxiety. Competitive activities (e.g., gaming) may increase facilitating anxiety [33]. Future research should develop tools and interventions to increase facilitating-type anxiety which will enhance student engagement, performance, as well as positively impact patient care.

The findings of objective 3 in this study suggest that nursing students with higher baseline anxiety levels are more prone to experiencing elevated debilitating anxiety during nursing simulation. This highlights the potential impact that reducing baseline levels of anxiety may have on reducing simulation anxiety and improving performance in stressful clinical scenarios. With this knowledge, educators can support students with high levels of debilitating anxiety through referrals to counseling services, ensuring opportunities for interpersonal connections, and using specific interventions that allow students to reflect on their sources of anxiety [34]. Moreover, nurse educators can instill a growth mindset through targeted simulation preparation activities that help all students better prepare for the specific patient scenario they will encounter in simulation [35].

Another notable finding of this study is that gender did not emerge as a significant factor in determining the type of anxiety experienced by nursing students. This suggests that the high anxiety levels in simulation contexts may not vary significantly between male and female students, highlighting the universality of simulation anxiety experienced by undergraduate nursing students. Previous research has reported higher levels of facilitating anxiety among males when compared to females [36,37], and females have been reported to have more overall anxiety than males due to psychosocial and biological factors [36,38].

As expected, the students with a diagnosed anxiety disorder exhibited significant differences in their baseline state and trait anxiety levels. This underscores the importance of individual differences and mental health factors when assessing anxiety nursing students in educational settings. Short-form instruments such as STAIS-5 and STAIT-5 provide easy, accessible ways for nursing educators to measure student anxiety levels, especially when anxiety is affecting a student’s performance in simulation or clinical settings [24]. Moreover, the recognition of individual differences, such as diagnosed anxiety disorders, underscores the importance of providing individualized support for students with mental health concerns. Implementing proactive measures, such as counseling services, stress management techniques, and accommodations, can help mitigate the impact of anxiety on student learning and performance [34].

The early identification of nursing students with high levels of debilitating-type anxiety will allow future testing of targeted interventions to enhance coping, well-being, and to prevent attrition. When students are self-aware of their levels of debilitating and facilitating anxiety, this knowledge may help them build mindfulness and resilience, important factors in the retention of nurses and preventing burnout [11]. Future research should use the AAT to investigate whether early identification and treatment for those with high debilitating anxiety can mitigate attrition from nursing programs. Since high rates of anxiety are closely related to stress and burnout in nursing, this has profound implications for nursing education, practice, and workforce retention.

### Limitations

This study has several limitations such as the use of convenience sampling at one site which limits the generalizability of these findings. Future studies should evaluate the AAT across multiple institutions with larger sample sizes. Additionally, this study only measured self-reported anxiety based on students’ own perceptions of their experiences with anxiety, and therefore, future studies should also examine students’ biometric markers for anxiety such as heart rate, cortisol, etc. for a multi-dimensional analysis of anxiety. Future research should include a confirmatory factor analysis with a larger sample and revised scale which eliminates or revises problematic items.

## 5. Conclusions

This study validates the revised AAT as a reliable instrument for measuring the levels of debilitating and facilitating anxiety in nursing simulation contexts, but future research is needed with larger samples and in diverse settings. Simulation anxiety involves complex and highly individualized factors, and this study highlights the importance of addressing nursing students’ anxiety to help them transition to successful nursing practice. There is a critical need for the development of targeted interventions and support strategies to mitigate debilitating anxiety and increase facilitating anxiety in nursing education. By recognizing and addressing anxiety, educators can better prepare students for the challenges of clinical practice, ultimately improving the quality of patient care in healthcare settings.

## Figures and Tables

**Table 1:** Student sample demographics.

Characteristic	Total (*n* = 90)
Age	Range 19–31; mean age = 20.9
Gender	Female = 82 (91.1%) Male = 6 (6.7%) Non-binary = 2 (2.2%)
Ethnicity	White/Caucasian = 75 (83.3%) Hispanic = 6 (6.7%) Black = 3 (3.3%) Asian = 4 (4.4%) Other = 2 (2.2%)
Sexuality	Straight/Heterosexual = 76 (84.4%) Gay/Lesbian = 3 (3.3%) Bisexual = 10 (11.1%)
Anxiety Diagnosis	Yes = 36 (40%) No = 54 (60%)

**Table 2:** EFA results for debilitating anxiety scale.

Scale Items and Number	Factor 1	Factor 2	Mean (SD)	Cronbach’s Alpha
			32.75 (5.92)	0.815
Forget questions or skills (7)	0.766			
More important equals worse (5)	0.690			
Upset when poorly prepared (4)	0.624			
Upset if don’t do well at beginning (19)	0.623			
Tired and don’t care (13)	0.552			
Nervousness (1)	0.549			
Time pressure causes to do worse (14)	0.542			
Mind goes blank (11)	0.495			
Fear of bad grade (3)	0.342			
Read without understanding (17)		−0.809		
Eigenvalues	3.90	1.016		
Explained Variance (%)	43.4%	11.3%		
Explained Variance (total)	54.7%			

**Table 3:** EFA results for facilitating anxiety scale.

Scale Items and Number	Factor 1	Factor 2	Factor 3	Mean (SD)	Cronbach’s Alpha
				25.01 (4.41)	0.716
Better than other people in sim (10)	0.652				
Focused during simulation (9)	0.583				
Nervousness helps do better (8)	0.463				
Enjoy challenging simulation (16)		−0.763			
Look forward to simulations (12)		−0.505			
More important equals better I do (18)		−0.380	0.330		
Work effectively under pressure (2)			0.730		
Forget to be nervous (6)	0.341		0.412		
Can cram (15)	No significant correlation				
Eigenvalues	3.08	1.209	1.14		
Explained Variance (%)	34.2%	13.4%	12.7%		
Explained Variance (total)	60.3%				

**Table 4:** Anxiety levels based on history of prior anxiety diagnosis.

Type of Anxiety	Yes Anxiety Diagnosis (*n* = 36) Mean (SD)	No Anxiety Diagnosis (*n* = 53) Mean (SD)	*p*-Value *
Debilitating	34.19 (6.07)	31.77 (5.60)	0.054 **
Facilitating	24.67 (5.35)	25.00 (3.83)	0.75 ***
Baseline State	11.54 (3.10)	9.23 (3.10)	<0.001 **
Baseline Trait	14.75 (3.29)	12.55 (3.48)	0.004 **
Pre-simulation State	13.18 (3.46)	11.81 (3.84)	0.097 **
Pre-simulation Trait	12.79 (3.56)	11.62 (3.39)	0.126 **

* Two-sided test of significance. ** Equal variances assumed. *** Equal variances not assumed.

**Table 5:** Correlation coefficients for anxiety.

	Debilitating Anxiety	Facilitating Anxiety
Baseline		
State	0.382 *	−0.428 *
Trait	0.462 *	−0.286 **
Pre-simulation		
State	0.552 *	−0.430 *
Trait	0.567 *	−0.415 *

* *p* < 0.001 ** *p* = 0.007.

## Data Availability

The data presented in this study are available on request by correspondence with the corresponding author.

## Public Involvement Statement

No public involvement in any aspect of this research.

## Guidelines and Standards Statement

This manuscript was drafted against the SQUIRE-EDU (Standards for Quality Improvement Reporting Excellence in Education: Publication Guidelines for Educational Improvement) for quantitative research.
